# Stimulating the *sir2–spargel* axis rescues exercise capacity and mitochondrial respiration in a *Drosophila* model of Barth syndrome

**DOI:** 10.1242/dmm.049279

**Published:** 2022-10-05

**Authors:** Deena Damschroder, Rubén Zapata-Pérez, Kristin Richardson, Frédéric M. Vaz, Riekelt H. Houtkooper, Robert Wessells

**Affiliations:** ^1^ Wayne State University School of Medicine, Department of Physiology, Detroit, MI 48201, USA; ^2^ Amsterdam UMC location University of Amsterdam, Department of Clinical Chemistry and Pediatrics, Laboratory Genetic Metabolic Diseases, Emma Children's Hospital, Meibergdreef 9, Amsterdam 1105AZ, The Netherlands; ^3^ Amsterdam Gastroenterology Endocrinology Metabolism Institute, Amsterdam 1105AZ, The Netherlands; ^4^ Core Facility Metabolomics, Amsterdam UMC location University of Amsterdam, Amsterdam 1105AZ, The Netherlands; ^5^ Amsterdam Cardiovascular Sciences Institute, Amsterdam 1105AZ, The Netherlands; ^6^ Emma Center for Personalized Medicine, Amsterdam UMC, Amsterdam 1105AZ, The Netherlands

**Keywords:** Nicotinamide riboside, NAD^+^, Barth syndrome, Exercise tolerance, *Drosophila*, *Tafazzin*

## Abstract

Cardiolipin (CL) is a phospholipid required for proper mitochondrial function. Tafazzin remodels CL to create highly unsaturated fatty acid chains. However, when *TAFAZZIN* is mutated, CL remodeling is impeded, leading to mitochondrial dysfunction and the disease Barth syndrome. Patients with Barth syndrome often have severe exercise intolerance, which negatively impacts their overall quality of life. Boosting NAD^+^ levels can improve symptoms of other mitochondrial diseases, but its effect in the context of Barth syndrome has not been examined. We demonstrate, for the first time, that nicotinamide riboside can rescue exercise tolerance and mitochondrial respiration in a *Drosophila Tafazzin* mutant and that the beneficial effects are dependent on *sir2* and *spargel*. Overexpressing *spargel* increased the total abundance of CL in mutants. In addition, muscles and neurons were identified as key targets for future therapies because *sir2* or *spargel* overexpression in either of these tissues is sufficient to restore the exercise capacity of *Drosophila Tafazzin* mutants.

## Teaser

**Summary:** Nicotinamide riboside rescues the exercise capacity and mitochondrial function of a *Drosophila* model of Barth syndrome in a Sir2/Spargel-dependent manner.

## INTRODUCTION

The mitochondrial-specific phospholipid cardiolipin (CL) has a substantial impact on mitochondrial metabolism. CL supports mitochondrial function in multiple ways, including by directly binding to the complexes of the respiratory chain (Fry and Green, 1980; Schlame and Haldar, 1993; Pfeiffer et al., 2003) and assisting with the formation of super complexes to allow efficient oxidative phosphorylation (Pfeiffer et al., 2003; McKenzie et al., 2006). CL is synthesized within the inner mitochondrial membrane, and *de novo* CL undergoes a remodeling process that alters the CL fatty acid composition to contain mostly unsaturated fatty acids (Hostetler et al., 1971; Schlame et al., 2005; Schlame, 2013).

Tafazzin is the primary enzyme that remodels CL, and loss-of-function mutations in *TAFAZZIN* increase the ratio of monolysocardiolipin (MLCL) to CL (Vreken et al., 2000). This disturbance of CL metabolism leads to mitochondrial dysfunction and a rare disease known as Barth syndrome (Barth et al., 1983; Vreken et al., 2000; Schlame et al., 2002). Barth syndrome is an X-linked disorder that is characterized by cardiomyopathy, neutropenia, muscle weakness, delayed growth, increased urinary 3-methylglutaconic aciduria and exercise intolerance (Neustein et al., 1979; Barth et al., 1983; Kelley et al., 1991; Roberts et al., 2012; Cade et al., 2017; Bittel et al., 2018), with patients reporting that their exercise intolerance is one of the principal symptoms to negatively impact their lives (https://fda.report/media/130562/EL-PFDD+Meeting+on+Barth+Syndrome+Voice+of+the+Patient+Report.pdf).

Barth syndrome currently has no cure, and patient care focuses on symptom management, making continued efforts to find treatments to reduce the severity of symptoms imperative. One possible therapeutic avenue is manipulating the levels of nicotinamide adenine dinucleotide (NAD^+^). A decreased NAD^+^ concentration is associated with many mitochondrial diseases, age-related diseases and general aging (Zakhary et al., 2010; Massudi et al., 2012; Guadalupe-Grau et al., 2017; Clement et al., 2019; Fang et al., 2019; Pirinen et al., 2020; Zapata-Perez et al., 2021). In various disease states, supplementation with NAD^+^ precursors such as nicotinamide riboside (NR), nicotinic acid (NA) and nicotinamide mononucleotide (NMN) provides improvements to mitochondrial function in flies (Lehmann et al., 2017) and in mice (Khan et al., 2014; Zhou et al., 2020), highlighting the importance of maintaining NAD^+^ homeostasis.

There are no major side effects reported with NR supplementation (Martens et al., 2018; Conze et al., 2019), although supplementation with NA can cause uncomfortable skin flushing (Kamanna et al., 2009). Both NMN and NR supplementation can increase NAD^+^ levels, but the oral bioavailability and transport into cells differs between the two compounds, with NR transported directly into the cell, and NMN either converted to NR by CD73 and then transported into the cell (Garavaglia et al., 2012; Grozio et al., 2013; Ratajczak et al., 2016; Rajman et al., 2018) or directly transported by SLC12A8 in the small intestine (Grozio et al., 2019). Chronic NR and NMN supplementation is well tolerated in humans (Conze et al., 2016, 2019; Trammell et al., 2016; Martens et al., 2018; Elhassan et al., 2019; Remie et al., 2020; Yoshino et al., 2021).

Sirtuins are known for their role in longevity and mitochondrial health (Li et al., 2008; Fang et al., 2016; Imai and Guarente, 2016; Lautrup et al., 2019; Zapata-Perez et al., 2021). Dietary supplementation with NMN or NR can increase the activity of SIR2 (also known as SIRT1) (Yoshino et al., 2011; Cantó et al., 2012; Wang et al., 2018). Among its many targets, SIR2 can deacetylate PGC-1α (also known as PPARGC1A) and increase its activity (Rodgers et al., 2005; Imai and Guarente, 2016). PGC-1α is a well-studied transcriptional co-activator that regulates the expression of mitochondrial oxidative phosphorylation genes to improve mitochondrial function and increase mitochondrial biogenesis (Wu et al., 1999; Lehman et al., 2000; Lai et al., 2014; Imai and Guarente, 2016). PGC-1α is upregulated with exercise training and upregulates antioxidant defense systems to limit oxidative damage from increased mitochondrial respiration (Handschin et al., 2007; Austin and St-Pierre, 2012).

The function of the *Drosophila Pgc-1α* (also known as *Ppargc1a*) homolog, *spargel* (*srl*), is similar to that of its mammalian counterpart, including its role in longevity, mitochondrial biogenesis, oxidative stress resistance and exercise adaptations (Tiefenböck et al., 2010; Rera et al., 2011; Tinkerhess et al., 2012b; Mukherjee et al., 2014). In the context of Barth syndrome, overexpressing either *sir2* (also known as *Sirt1*) or *spargel* in a *Drosophila Tafazzin* mutant can alter the CL profile towards wild-type levels (Xu et al., 2019). However, the impact of these changes on exercise tolerance and mitochondrial respiration has not previously been examined.

Here, we demonstrate, for the first time, the beneficial effects of NR administration in the context of Barth syndrome. Supplementation with NR was sufficient to restore the endurance and mitochondrial function of *Drosophila Tafazzin* mutants. We further show that these beneficial effects require *sir2* and *spargel*, and that overexpressing *sir2* or *spargel* can rescue the exercise phenotypes of *Drosophila Tafazzin* mutants. Overexpression of *spargel* can reduce the MLCL:CL ratio. Finally, we demonstrate that muscle and neurons are key targets for future Barth syndrome therapies.

## RESULTS

### *Taz^889^* flies have similar phenotypes to Barth patients

**Fig. 1.**
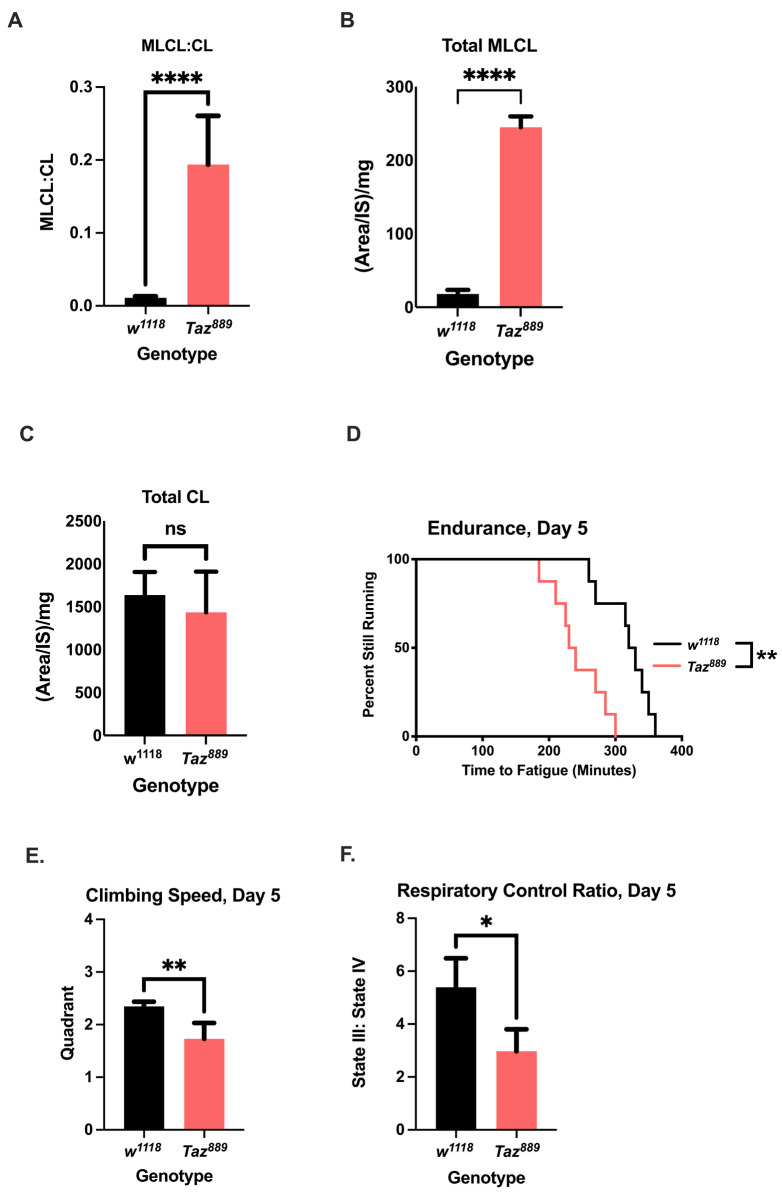
***Taz^889^* has similar phenotypes to Barth patients.** (A) The monolysocardiolipin (MLCL):cardiolipin (CL) ratio in *Taz^889^* flies is significantly increased compared to that in controls (*w^1118^*) (unpaired two-tailed Student's *t-*test, six biological repetitions with six flies per repetition). (B,C) *Taz^889^* flies have an increased abundance of total MLCL compared to controls (B), but there is no difference in total CL (C) (unpaired two-tailed Student's *t-*test, six biological repetitions with six flies per repetition). (D) *Taz^889^* flies have reduced endurance compared to that of controls (*n*=8 vials,160 flies total, log-rank analysis). (E) *Taz^889^* flies have a reduced climbing speed compared to that of controls (unpaired two-tailed Student's *t*-test, *n*=100 flies, data are mean±s.d.). (F) The respiratory control ratio of isolated mitochondria from *Taz^889^* flies is significantly reduced compared to that of controls (unpaired two-tailed Student's *t*-test, six biological replicates, *n*=60 flies per replicate, data are mean±s.e.m.). *P<0.05, **P<0.01, ****P<0.0001; ns, not significant.

A new mutant allele (*Taz^889^*) was generated that has the same lesion as a previously published allele (Xu et al., 2006), but with a red fluorescent protein (RFP) marker knocked in to facilitate the introduction of additional transgenic elements (Fig. S1). To confirm that the new allele retained canonical Tafazzin mutant phenotypes, we examined the lipid profile and the exercise capacity of *Taz^889^* flies, because both are altered when Tafazzin function is reduced (Neuwald, 1997; Vreken et al., 2000; Xu et al., 2006; Houtkooper et al., 2009a; Schlame, 2013; Damschroder et al., 2018a). The ratio of total MLCL to total CL, which is the prime diagnostic marker for Barth syndrome (Kulik et al., 2008; Houtkooper et al., 2009b; Molenaars et al., 2021), was increased in *Taz^889^* flies compared to that in control flies (*w^1118^*) (Fig. 1A, unpaired two-tailed Student's *t*-test, *P*=0.0002), with the total abundance of MLCL being higher in *Taz^889^* flies than in controls (Fig. 1B, unpaired two-tailed Student's *t*-test, *P*<0.0001). The total amount of CL was not different between *Taz^889^* flies and control flies (Fig. 1C, unpaired two-tailed Student's *t*-test, *P*=0.386). The most abundant CL species in control flies (*w^1118^*) were 64:4 and 66:5, and these were specifically reduced in *Taz^889^* flies (Fig. S2A). The most abundant MLCL species to accumulate in *Taz^889^* flies was 48:3 (Fig. S2B).

The exercise capacity of *Taz^889^* mutants was assessed by measuring their endurance on the *Drosophila* exercise platform known as the Power Tower (Tinkerhess et al., 2012a; Damschroder et al., 2018b). Similar to the Δ*Taz* allele (Xu et al., 2006), *Taz^889^* flies had reduced endurance (Fig. 1D, log-rank, *P*=0.0014) and reduced climbing speed (Fig. 1E, unpaired two-tailed Student's *t*-test, *P*=0.0078) compared to those of controls (Damschroder et al., 2018a). To test mitochondrial function in mutants, the respiratory control ratio (RCR) of isolated mitochondria was measured. The RCR is the ratio of state III respiration (ADP stimulated) to state IV respiration (oligomycin stimulated) and reflects general mitochondrial health (Gonzalvez et al., 2013). Mutants had a reduced RCR, indicating that their mitochondria had reduced efficiency of mitochondrial coupling (Fig. 1F, unpaired two-tailed Student's *t*-test, *P*=0.038). Taken together, these results demonstrate that the *Taz^889^* allele retains stereotypical phenotypes of reduced Tafazzin function.

### NR supplementation rescues the exercise capacity of *Taz^889^* flies

**Fig. 2.**
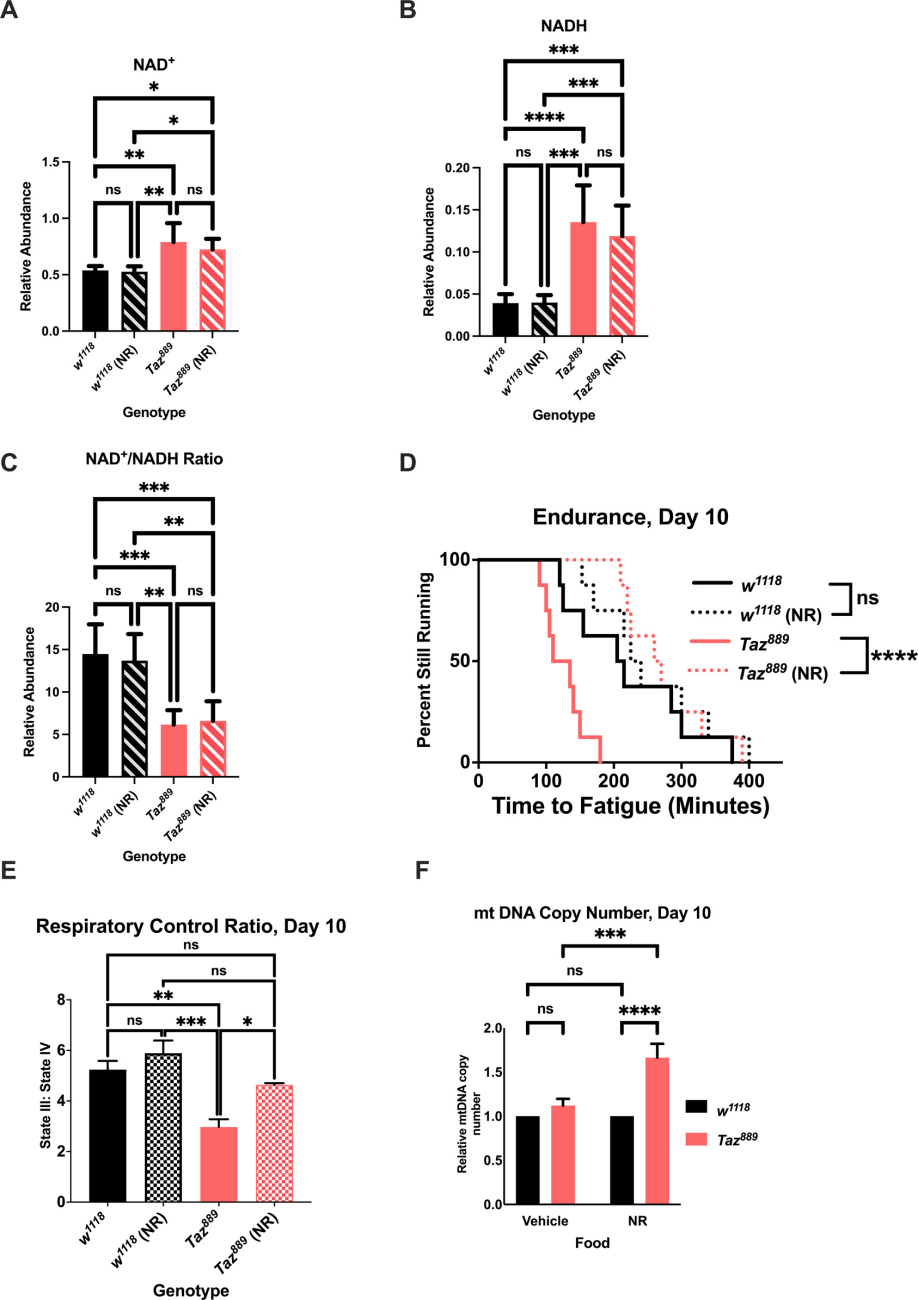
**Nicotinamide riboside (NR) supplementation provides benefits to *Tafazzin* mutants.** (A,B) The abundance of NAD^+^ (B) and NADH (A) was measured by mass spectrometry and normalized to total protein levels (six biological repetitions with six flies per repetition, data are mean±s.d., two-way ANOVA, genotype effect, *P*<0.0001, Tukey post-hoc test). (C) The NAD^+^:NADH ratio was calculated from the abundances for those molecules (data are mean±s.d., two-way ANOVA, genotype effect, *P*<0.0001, Tukey post-hoc test). (D,E) NR supplementation restores the endurance of *Taz^889^* flies (log-rank analysis, *n*=8 vials, 20 flies per vial; D) and the respiratory control ratio (RCR) (six biological replicates, data are mean±s.e.m., *n*=60 per replicate, genotype effect, *P*<0.0001, NR effect, *P*=0.0029, Tukey post-hoc test; E). (F) At day 10, the relative mitochondrial DNA (mtDNA) copy number is not different between *Taz^889^* and control flies, but NR supplementation increases mtDNA in *Taz^889^* flies (*n*=3 biological replicates, data are mean±s.d., two-way ANOVA, genotype effect, *P*<0.0001, NR effect, *P*=0.0006, Tukey post-hoc test). (G) Feeding NR to *Taz^889^* flies does not alter the MLCL:CL ratio (unpaired two-tailed Student's *t-*test, six biological repetitions with six flies per repetition). (H,I) There is no difference in total MLCL (H) between *Taz^889^* flies with and without NR feeding, but NR-fed *Taz^889^* flies have an increased abundance of total CL (I) (unpaired two-tailed Student's *t-*test, six biological repetitions with six flies per repetition). *P<0.05, **P<0.01, ***P<0.001, ****P<0.0001; ns, not significant.

*Taz^889^* flies had a higher abundance of NAD^+^ and NADH than control flies from the same genetic background (*w^1118^*) (Fig. 2A, two-way ANOVA, Tukey post-hoc test, *P*=0.0017), but the NAD^+^:NADH ratio was significantly reduced in the mutants (Fig. 2C, two-way ANOVA, Tukey post-hoc test, *P*=0.0008). NR supplementation at various concentrations resulted in improvements to endurance, with 1 mM generating the largest improvement to endurance (Fig. S3A). The NR concentration in the food did not affect the feeding rate (Fig. S3B, two-way ANOVA, *P*=0.08). NR supplementation did not significantly change the levels of NADH (Fig. 2A, two-way ANOVA, Tukey post-hoc test, *P*=0.772), NAD^+^ (Fig. 2B, two-way ANOVA, Tukey post-hoc test, *P*=0.688) or the NAD^+^:NADH ratio between NR-fed mutants and vehicle-fed mutants (Fig. 2C, two-way ANOVA, Tukey post-hoc test, *P*=0.990).

However, the endurance of NR-fed mutants increased after 5 days of supplementation (Fig. 2D, log-rank, *P*<0.0001), although not after 3 days of supplementation (Fig. S3C, log-rank, *P*=0.487). The RCR of NR-fed mutants was also rescued to control levels (Fig. 2E, two-way ANOVA, Tukey post-hoc test, *P*=0.607). NR supplementation provided no benefit to the control line in endurance or RCR (Fig. 2E, two-way ANOVA, Tukey post-hoc test, *P*=0.544). To determine whether there was a difference in mitochondrial number, the mitochondrial DNA (mtDNA) copy number was measured using the relative expression of a mitochondrial gene, *mitochondrial large ribosomal RNA* (*lrRNA*; also known as *mt:lrRNA*), to that of a nuclear gene, *RNA polymerase II* (*rp2*; also known as *Polr2F*) (Correa et al., 2012). NR-fed mutants exhibited an increase in mtDNA copy number relative to vehicle-fed mutants (Fig. 2F, two-way ANOVA, Tukey post-hoc test, *P*=0.0003), whereas NR-fed control flies did not have more mitochondria relative to vehicle-fed controls (Fig. 2F, two-way ANOVA, Tukey post-hoc test, *P*=0.978). After NR feeding, the MLCL:CL ratio was not reduced in *Taz^889^* flies (Fig. 2G, unpaired two-tailed Student's *t*-test, *P*=0.130) and the total MLCL content was not different (Fig. 2H, unpaired two-tailed Student's *t*-test, *P*=0.069). The total CL content was elevated in *Taz^889^* NR-fed flies (Fig. 2I, unpaired two-tailed Student's *t*-test, *P*=0.033). Thus, NR supplementation to mutants can restore endurance, improve mitochondrial function and increase mitochondrial number, without measurably changing total NAD^+^ and NADH content or the NAD^+^:NADH ratio.

**Fig. 3.**
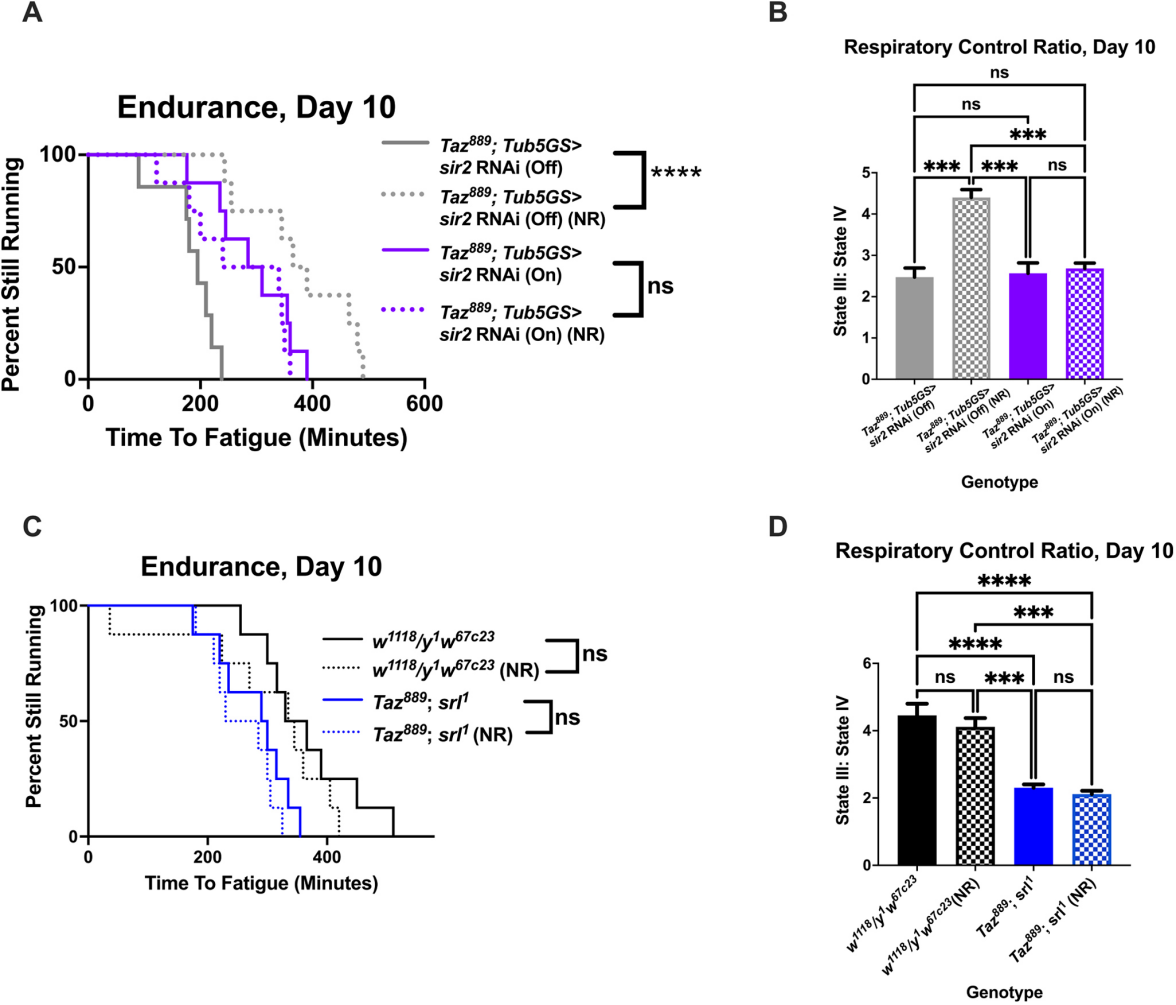
**The beneficial effects of NR are *sir2* and *spargel* (*srl*) dependent*.*** (A) When *sir2* is knocked down in *Taz^889^* flies, NR supplementation does not improve their endurance (*Taz^889^; Tub5GS>uas-sir2* RNAi On) (log-rank analysis, *n*=8 vials, 20 flies per vial). (B) The RCR is not restored by NR supplementation when *sir2* is knocked down in *Taz^889^* flies (six biological replicates, data are mean±s.e.m., *n*=60 flies per biological replicate, two-way ANOVA, genotype effect, *P*=0.002, Tukey post-hoc test). (C) When the *Drosophila Pgc-1α* homolog *spargel* is mutated (*srl^1^*) in *Taz^889^* flies, NR feeding does not improve the endurance of *Taz^889^* flies (log-rank analysis, *n*=8 vials, 20 flies per vial). (D) The RCR of the double mutant (*Taz^889^; srl^1^*) is not improved after NR supplementation (six biological replicates, data are mean±s.e.m., *n*=60 flies per replicate, two-way ANOVA, genotype effect, *P*<0.0001, Tukey post-hoc test). ***P<0.001, ****P<0.0001; ns, not significant. On, mifepristone-induced gene expression; Off, no mifepristone-induced gene expression.

A possible reason for the levels of NAD^+^ and NADH, and the NAD^+^:NADH ratio not changing with NR supplementation is that excess NAD^+^ is rapidly utilized to induce the rescue phenotype (Fang et al., 2017). A possible consumer of NAD^+^ that could be responsible for the rescue phenotype is *sir2*, which is an NAD^+^-dependent deacetylase (Stein and Imai, 2012). SIR2 has many targets, including the well-studied exercise protein PGC-1α (Baar et al., 2002; Rodgers et al., 2005; Handschin et al., 2007; Olesen et al., 2010; Tinkerhess et al., 2012b). Therefore, we hypothesized that *sir2* and *spargel* are required for the rescue phenotypes observed with NR supplementation. Using mifepristone-inducible gene-switch lines, we induced whole-body changes to gene expression in an adult-specific manner. Gene expression was confirmed using quantitative reverse transcription PCR (qRT-PCR) (Fig. S4). When *sir2* was knocked down in *Taz^889^* flies, NR no longer increased endurance (Fig. 3A, log-rank, *P*=0.432) or improved RCR (Fig. 3B, two-way ANOVA, Tukey post-hoc test, *P*=0.982). Likewise, when *spargel* was mutated in *Taz^889^* flies, NR supplementation did not increase the endurance (Fig. 3C, log-rank, *P*=0.25) or the RCR (Fig. 3D, two-way ANOVA, Tukey post-hoc test, *P*=0.941). Taken together, these results confirm that *sir2* and *spargel* are required for NR supplementation to increase endurance or RCR.

### Overexpressing *sir2* or *spargel* is sufficient to restore the endurance of *Taz^889^* flies

**Fig. 4.**
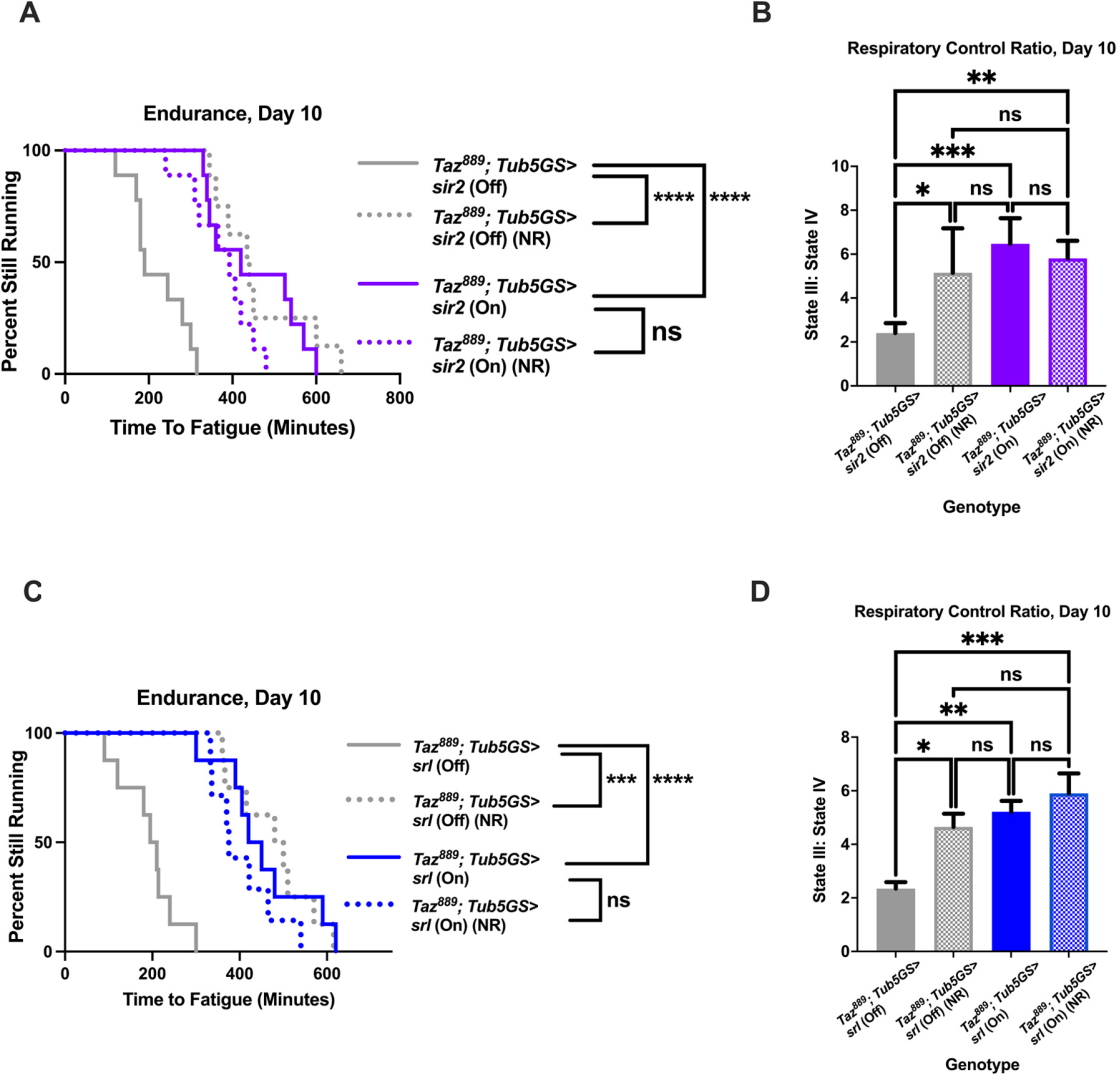
**Overexpression of *sir2* or *srl* is sufficient to improve the exercise capacity of *Taz^889^* flies*.*** (A,B) Overexpressing *sir2* in *Taz^889^* flies (*Taz^889^;Tub5GS>uas-sir2* On) increases their endurance (*n*=8 vials, 160 flies total, pair-wise log rank analysis; A) and improves the RCR (six biological replicates, data are mean±s.e.m., *n*=60 flies per replicate, two-way ANOVA, genotype effect, *P*=0.0002, Tukey post-hoc test; B). NR feeding does not provide further improvements to these phenotypes. (C,D) The endurance (*n*=8 vials, 160 flies total, pair-wise log rank analysis; C) and RCR (six biological replicates, data are mean±s.e.m., *n*=60 flies per replicate, two-way ANOVA, genotype effect, *P*=0.0002, NR effect, *P*=0.045, Tukey post-hoc test; D) of *Taz^889^* flies overexpressing *srl* (*Taz^889^; Tub5GS>uas-srl* On) are increased compared to those of *Taz^889^* flies without *srl* overexpression, but supplementation with NR does not provide any additive benefits. *P<0.05, **P<0.01, ***P<0.001; ns, not significant. On, mifepristone-induced gene expression; Off, no mifepristone-induced gene expression.

We next hypothesized that overexpressing *sir2* or *spargel* would be sufficient to restore endurance to *Taz^889^* flies. The endurance of mutants was increased when *sir2* was overexpressed (Fig. 4A, log-rank, *P*<0.0001) and there was no additive effect with NR supplementation (Fig. 4A, log-rank, *P*=0.093). The RCR of mutants was also improved with *sir2* overexpression (Fig. 4B, two-way ANOVA, Tukey post-hoc test, *P*=0.0005), but NR supplementation did not provide further improvements (Fig. 4B, two-way ANOVA, Tukey post-hoc test, *P*=0.834). Overexpression of *spargel* in mutants produced similar results, causing an increase in endurance (Fig. 4C, log-rank, *P*<0.0001) and the RCR (Fig. 4D, two-way ANOVA, Tukey post-hoc test, *P*=0.002). There were no additive improvements to the endurance (Fig. 4C, log-rank, *P*=0.23) or the RCR (Fig. 4D, two-way ANOVA, Tukey post-hoc test, *P*=0.713) with NR supplementation. Additionally, no additive effects were observed with NR supplementation to control flies overexpressing *sir2* or *spargel* (Fig. S5).

### Overexpressing *spargel* in *Taz^889^* flies improves the NAD^+^:NADH ratio, increases mtDNA and reduces the MLCL:CL ratio

**Fig. 5.**
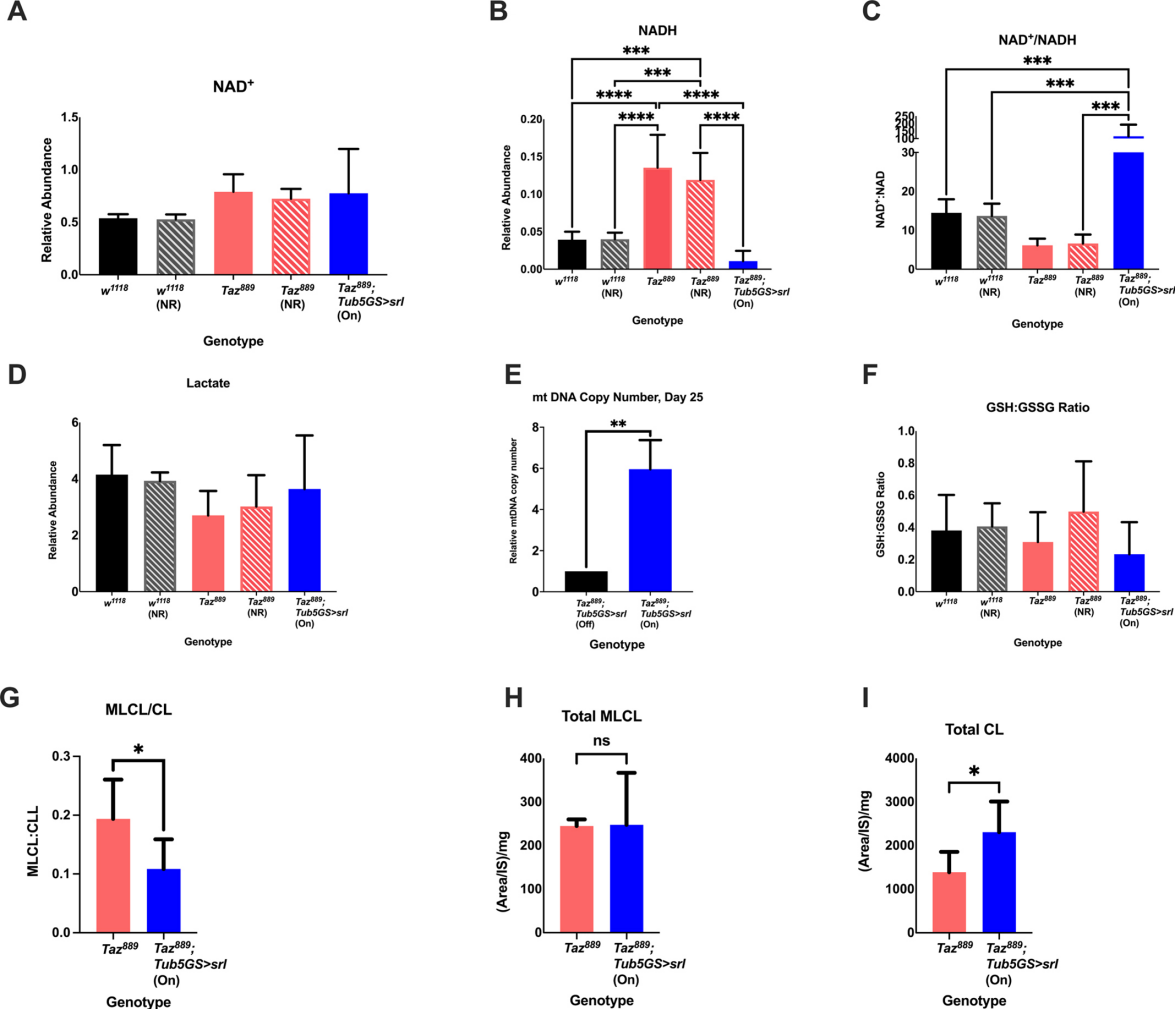
**Overexpressing *srl* in *Taz^889^* flies improves the NAD^+^:NADH ratio, increases the mtDNA copy number and reduces the MLCL:CL ratio.** All measurements were performed on flies at day 10, except the measurement of mtDNA copy number, which was performed at day 25. The abundance of NAD^+^ and NADH was measured by mass spectrometry and normalized to total protein levels (six biological repetitions with ten flies per repetition, data are mean±s.d., one-way ANOVA, Tukey post-hoc test). (A) There is no significant difference in the relative abundance of NAD^+^ between control and *Taz^889^* flies with and without NR feeding, or *Taz^889^* flies with and without overexpression of *srl* (one-way ANOVA, *P*=0.096). (B,C) Overexpressing *srl* in *Taz^889^* flies significantly reduces NADH relative to all other groups (B), consequently increasing the NAD^+^:NADH ratio (C). (D) There is no difference in lactate production between experimental groups (one-way ANOVA, *P*=0.194). (E) mtDNA copy number represents the relative expression of the mitochondrial gene *lrRNA* to the nuclear gene *rp2*. At day 25, *Taz^889^* flies overexpressing *srl* have a higher mtDNA copy relative to that of *Taz^889^* flies (*n*=3 biological replicates, data are mean±s.d., unpaired two-tailed Student's *t-*test). (F) The ratio of glutathione (GSH) to glutathione disulfide (GSSG) is not significantly different between groups (one-way ANOVA, *P*=0.316). (G) The MLCL:CL ratio of *Taz^889^* flies overexpressing *srl* is significantly reduced relative to that of *Taz^889^* flies (unpaired two-tailed Student's *t*-test, three biological replicates, *n*=6 flies per biological replicate, data are mean±s.d.). (H,I) The reduced MLCL:CL ratio is not due to a reduced total MLCL amount (unpaired two-tailed Student's *t*-test, three biological replicates, *n*=6 flies per biological replicate, data are mean±s.d., *P*=0.954; H), but to an increased total CL amount (unpaired two-tailed Student's *t*-test, three biological replicates, *n*=6 flies per biological replicate, data are mean±s.d.; I). *P<0.05, **P<0.01, ***P<0.001, ****P<0.0001; ns, not significant. On, mifepristone-induced gene expression; Off, no mifepristone-induced gene expression.

To determine whether overexpression of *spargel* in *Taz^889^* flies influenced the NAD^+^:NADH ratio, we measured the relative abundance of NAD^+^ and NADH. There was no difference in the abundance of NAD^+^ (Fig. 5A). *Taz^889^* flies overexpressing *spargel* had a lower NADH abundance than mutants (Fig. 5B, two-way ANOVA, Tukey post-hoc test, *P*<0.0001) or *w^1118^* (control) flies (Fig. 5B, two-way ANOVA, Tukey post-hoc test, *P*=0.0002), resulting in a higher NAD^+^:NADH ratio (Fig. 5C, two-way ANOVA, Tukey post-hoc test, *P*<0.0001). The reduction in NADH was not due to increased lactate production (Fig. 5D), suggesting a possible effect at the mitochondrial level. To determine whether there was an increase in mitochondrial biogenesis, the mtDNA copy number was measured. *Taz^889^* flies overexpressing *spargel* showed an increase in mtDNA copy number (Fig. 5E, unpaired two-tailed Student's *t*-test, *P*=0.0012), but there were no differences in the glutathione (GSH) to glutathione disulfide (GSSG) ratio (Fig. 5F, two-way ANOVA, *P*=0.316), indicating that there was no increase in oxidative stress. The MLCL:CL ratio in *Taz^889^* flies overexpressing *spargel* was significantly reduced compared to that in *Taz^889^* flies without *spargel* overexpression (Fig. 5G, unpaired two-tailed Student's *t*-test, *P*=0.032). *Taz^889^* flies with *spargel* overexpression showed increased total CL (Fig. 5I, unpaired two-tailed Student's *t*-test, *P*=0.023), without an increase in the total amount of MLCL (Fig. 5H, unpaired two-tailed Student's *t*-test, *P*=0.945).

### Tafazzin is required in both muscle and neurons for normal endurance

Patients with Barth syndrome often display exercise intolerance due to their skeletal myopathy, low muscle tone and cardiomyopathy (Barth et al., 1983; Spencer et al., 2006; Thompson et al., 2016). However, the tissue-specific requirements for *TAFAZZIN* for normal exercise capacity have not been rigorously investigated. We wanted to identify the key tissues responsible for the exercise intolerance of *Tafazzin* mutants as a first step to investigating whether tissue-specific expression of *sir2* or *spargel* could rescue endurance and mitochondrial function. Prior to tissue-specific knockdown experiments, a whole-body driver was used to validate the RNA interference (RNAi) line, and, like the genomic mutant, ubiquitous knockdown of *Tafazzin* caused reduced endurance (Fig. S6A, log-rank, *P*=0.0006), climbing speed (Fig. S6B, unpaired two-tailed Student's *t*-test, *P*=0.012) and RCR (Fig. S6C, unpaired two-tailed Student's *t*-test, *P*<0.0001).

**Fig. 6.**
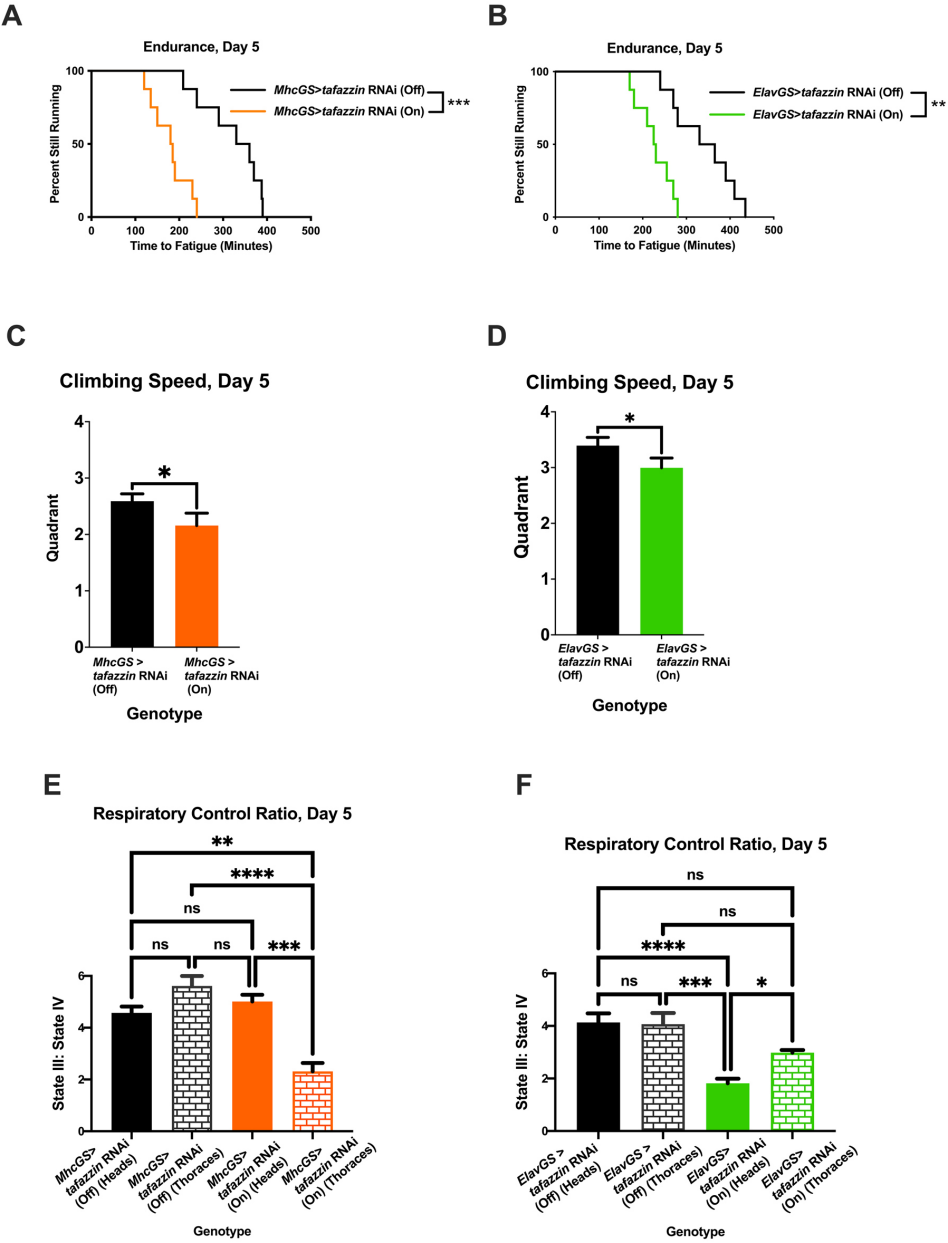
***Tafazzin* is required in either muscle or neurons for normal exercise capacity.** (A,B) Knocking down *Tafazzin* in muscle (A) or neuronal tissue (B) reduces endurance (log-rank analysis, *n*=8 vials, 20 flies per vial). (C,D) These tissue-specific knockdown flies have a reduced climbing speed (unpaired two-tailed Student's *t*-test, *n*=100 flies, data are mean±s.d.). (E,F) The RCRs of the muscle-enriched thoraces (two-way ANOVA, genotype effect, *P*=0.0002, tissue effect, *P*=0.013, Tukey post-hoc test; E) or the neuronal-enriched heads (two-way ANOVA, genotype effect, *P*<0.0001, tissue effect, *P*<0.0001, Tukey post-hoc test; F) are reduced relative to background controls and counter body part (data are averages of six biological replicates ±s.e.m., *n*=60 flies per replicate). *P<0.05, **P<0.01, ***P<0.001, ****P<0.0001; ns, not significant. On, mifepristone-induced gene expression; Off, no mifepristone-induced gene expression.

In the fly, *Tafazzin* is required in both muscle (Fig. 6A, log-rank, *P*=0.004) and neurons (Fig. 6B, log-rank, *P*=0.016) for normal endurance, but knocking down *Tafazzin* in the heart or fat body caused no significant reduction in endurance (Fig. S6D, log-rank, *P*=0.113; Fig. S6E, log-rank, *P*=0.2134). Climbing speed was also significantly reduced with muscle-specific and neuron-specific knockdown (Fig. 6C, unpaired two-tailed Student's *t*-test, *P*=0.0149; Fig. 6D, unpaired two-tailed Student's *t*-test, *P*=0.013). To examine the mitochondrial function of the tissue-specific knockdown flies, we isolated mitochondria from heads, which are enriched with neurons, and from the thoraces, which are enriched with muscle tissue. The RCR of mitochondria from the thoraces was reduced in the muscle-specific *Tafazzin* knockdown flies (Fig. 6E, two-way ANOVA, Tukey post-hoc test, *P*=0.002), and the RCR of mitochondria from heads was reduced in the neuron-specific *Tafazzin* knockdown flies (Fig. 6F, two-way ANOVA, Tukey post-hoc test, *P*=0.048). To confirm the specificity of the tissue-specific drivers, the expression level of *Tafazzin* was measured in the heads and thoraces of these tissue-specific knockdown flies. Muscle-specific *Tafazzin* knockdown flies had reduced *Tafazzin* expression in their thoraces relative to their heads, and the neuronal-specific *Tafazzin* knockdown flies had reduced *Tafazzin* expression in their heads relative to their thoraces (Fig. S4B), validating the specificity of the tissue drivers.

**Fig. 7.**
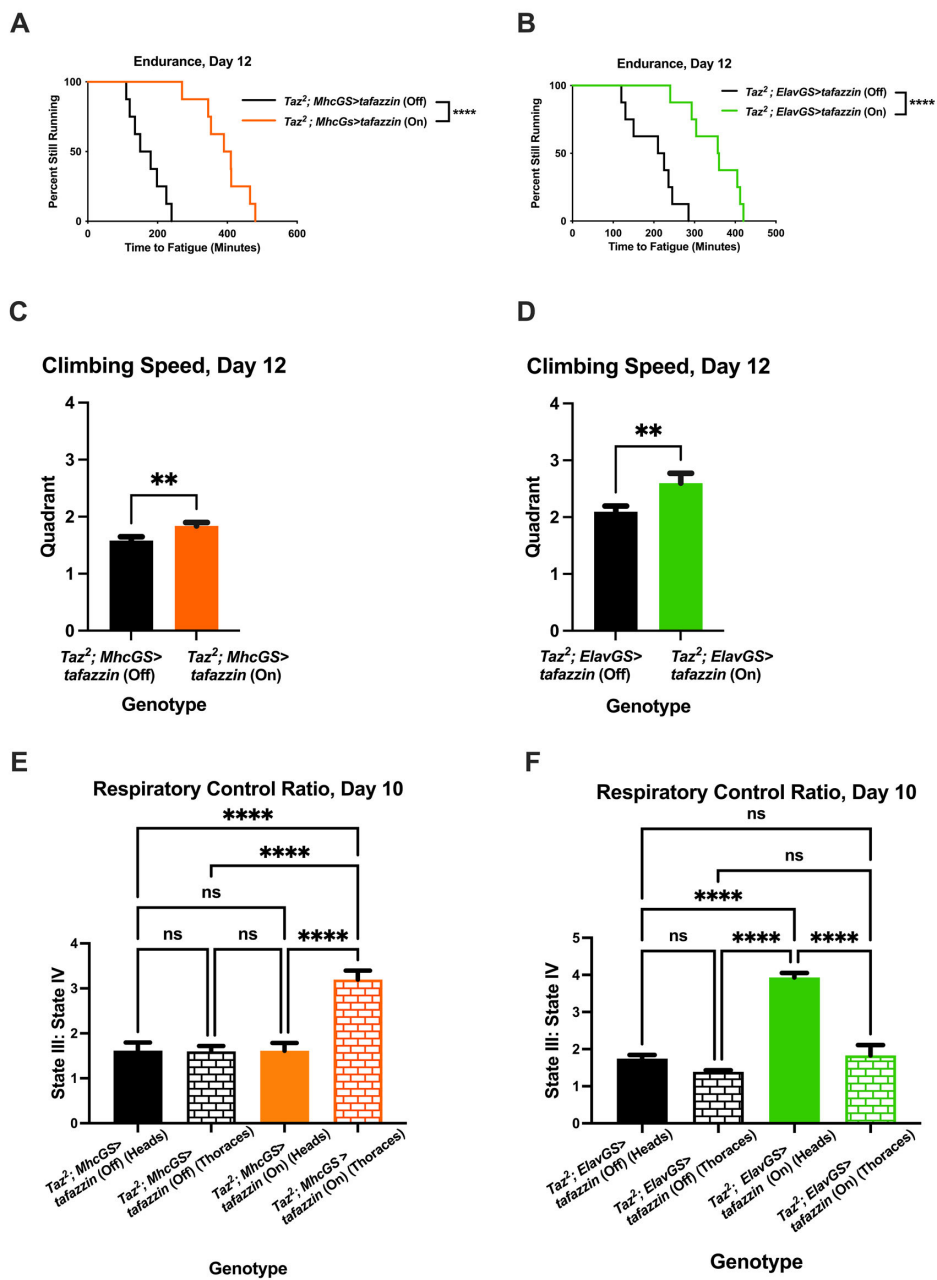
**Restoring *Tafazzin* in either muscle or neurons is sufficient to increase exercise capacity.** (A,B) When *Tafazzin* is restored in *Taz^2^* flies in either muscle (A) or neurons (B), the endurance is significantly increased by day 12 (log-rank analysis, *n*=8 vials, 20 flies per vial). (C,D) The climbing speed is also improved by day 12 (unpaired two-tailed Student's *t*-test, *n*=100 flies, data are mean±s.d.). (E,F) The RCR is increased in mitochondria isolated from the thoraces of the muscle-specific rescue flies (two-way ANOVA, genotype effect, *P*=0.0001, tissue effect, *P*=0.0002, Tukey post-hoc test; E) and in mitochondria isolated from the heads of the neuronal-specific rescue flies (two-way ANOVA, genotype effect, *P*<0.0001, tissue effect, *P*<0.0001, Tukey post-hoc test; F) (data are averages of six biological replicates ±s.e.m., *n*=60 per replicate). **P<0.01, ****P<0.0001; ns, not significant. On, mifepristone-induced gene expression; Off, no mifepristone-induced gene expression.

We next tested whether overexpressing *Tafazzin* in the muscle or neurons of *Taz^889^* flies would be beneficial. The endurance of the muscle-specific and the neuronal-specific rescue of *Tafazzin* was not significantly improved at day 5 (Fig. S7A, log-rank, *P*=0.679; Fig. S7B, log-rank, *P*=0.378), but by day 12 the endurance was higher (Fig. 7A, log-rank, *P*<0.0001; Fig. 7B, log-rank, *P*<0.0001). The climbing speed of the muscle-specific rescue flies and neuronal-specific rescue flies was also improved at day 12 (Fig. 7C, unpaired two-tailed Student's *t*-test, *P*=0.0013; Fig. 7D, unpaired two-tailed Student's *t*-test, *P*=0.0023), along with the RCR from both rescue types (Fig. 7E, two-way ANOVA, Tukey post-hoc test, *P*<0.0001; Fig. 7F, two-way ANOVA, Tukey post-hoc test, *P*<0.0001). The relative abundance of *Tafazzin* was not significantly different between day 5 and day 12 for the muscle-specific or neuronal-specific rescue flies (Fig. S4C, *P*=0.982, *P*=0.998), so the rescue phenotype at day 12 was not due to increased *Tafazzin* expression. These results confirm muscle and neurons as a key target for future Barth treatments.

### Tissue-specific overexpression of *sir2* and *spargel* improves endurance of *Taz^889^* flies

**Fig. 8.**
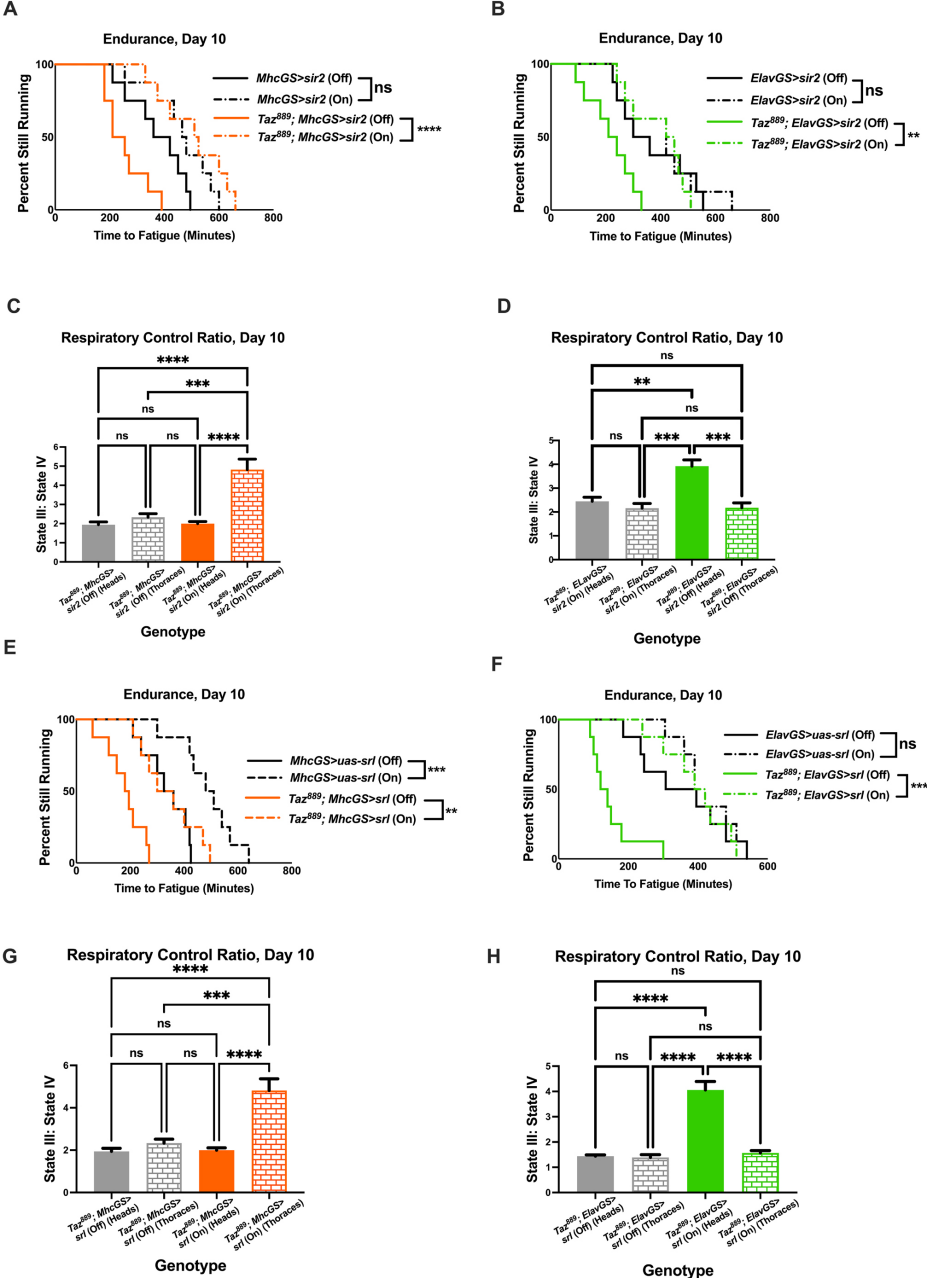
**Tissue-specific expression of *sir2* or *srl* causes beneficial phenotypes in *Taz^889^* flies.** (A,B) Overexpressing *sir2* in muscle (A) or neurons (B) of *Taz^889^* flies restores their endurance (log-rank analysis, *n*=8 vials, 20 flies per vial). (C) Muscle-specific overexpression of *sir2* restores the RCR of mitochondria isolated from thoraces (two-way ANOVA, genotype effect, *P*<0.0001, tissue effect, *P*=0.0005, Tukey post-hoc test) (data are averages of six biological replicates ±s.e.m., *n*=60 flies per replicate). (D) Neuronal-specific overexpression of *sir2* improves the RCR of mitochondria isolated from heads (two-way ANOVA, genotype effect, *P*=0.0001, tissue effect, *P*=0.0021, Tukey post-hoc test) (data are averages of six biological replicates ±s.e.m., *n*=60 flies per replicate). (E,F) *srl* overexpressed in muscle (E) or neurons (F) improves the endurance of *Taz^889^* flies (log-rank analysis, *n*=8 vials, 20 flies per vial). (G) Muscle-specific overexpression of *srl* increases the RCR of mitochondria from thoraces (two-way ANOVA, genotype effect, *P*<0.0001, tissue effect, *P*=0.0005, Tukey post-hoc test) (data are averages of six biological replicates ±s.e.m., *n*=60 flies per replicate). (H) Overexpressing *srl* in neurons improves the RCR of mitochondria isolated from heads (two-way ANOVA, genotype effect, *P*<0.0001, tissue effect, *P*<0.0001, Tukey post-hoc test) (data are averages of six biological replicates±s.e.m., *n*=60 flies per replicate). **P<0.01, ***P<0.001, ****P<0.0001; ns, not significant. On, mifepristone-induced gene expression; Off, no mifepristone-induced gene expression.

Overexpressing *sir2* or *spargel* ubiquitously in *Taz^889^* flies provided substantial benefits (Fig. 3). We hypothesized that overexpressing these genes in just muscle or neurons of mutants could be sufficient to rescue their endurance. *sir2* overexpression in the muscle or neurons of mutants rescued their endurance (Fig. 8A, log-rank, *P*<0.0001; Fig. 8B, log-rank, *P*=0.0034). When *sir2* was expressed in muscle, mitochondrial RCR was increased in thoraces but not in the heads (Fig. 8C, two-way ANOVA, Tukey post-hoc test, *P*<0.0001). When *sir2* was expressed in neurons, mitochondrial RCR was increased in heads but not thoraces (Fig. 8D, two-way ANOVA, Tukey post-hoc test, *P*=0.0003), which is consistent with a tissue-autonomous effect of Sir2.

Overexpressing *spargel* had a similar effect in both tissues. The endurance of *Taz^889^* flies was improved when *spargel* was expressed in muscle (Fig. 8E, log-rank, *P*=0.0017) or neurons (Fig. 8F, log-rank, *P*=0.0002). The RCR of mitochondria isolated from the thoraces of *Taz^889^* flies overexpressing *spargel* in their muscle was improved, whereas that of mitochondria isolated from the heads was not (Fig. 8G, two-way ANOVA, Tukey post-hoc test, *P*<0.0001). By contrast, overexpressing *spargel* pan-neuronally in *Taz^889^* flies improved the RCR of mitochondria isolated from heads, but not thoraces (Fig. 8H, two-way ANOVA, Tukey post-hoc test, *P*<0.0001), which is again consistent with a tissue-autonomous effect.

## DISCUSSION

When the remodeling process of CL is disturbed by *TAFAZZIN* mutations, the structural changes to the mitochondrial membrane cause reduced mitochondrial function and Barth syndrome (Neustein et al., 1979; Schlame et al., 2002). Although there is currently no cure for Barth syndrome, *Tafazzin* replacement therapy using adeno-associated virus (AAV) vector shows promising results in *Tafazzin* knockdown mice, including improved mitochondrial structure, mitochondrial respiration and heart function (Suzuki-Hatano et al., 2019). However, AVV gene therapy as a cure for Barth syndrome does not yet have US Food and Drug Administration (FDA) approval and not all patients have access to facilities capable of administering the treatment. Therefore, it is important for other therapeutic avenues to be examined.

Declining NAD^+^ abundance is a hallmark of mitochondrial diseases (Srivastava, 2016). Restoring NAD^+^ content in various disease models provides numerous benefits, including improved mitochondrial function (Yoshino et al., 2011; Cantó et al., 2012; Schondorf et al., 2018). In *Taz^889^* flies, the NAD^+^:NADH ratio was reduced. We demonstrate, for the first time, that NR supplementation is sufficient to rescue the exercise capacity and mitochondrial coupling of *Taz^889^* flies, despite causing no evident accumulation of NAD^+^ or changes in the NAD^+^:NADH ratio. NR supplementation provided no benefits to control flies, which supports the idea that NR supplementation is compensating for a deficit specific to *Taz^889^* flies and is not a general booster to endurance.

Like our results, one study using the genetic mitochondrial disease mouse model (*Sco2* knockout/knock-in model) found that NR supplementation improved the mutant's exercise capacity whereas their control group gained no benefits (Cerutti et al., 2014). Multiple studies demonstrate that benefits from NR or NA supplementation work through SIR2 (Cerutti et al., 2014; Khan et al., 2014; Pirinen et al., 2020), further supporting the findings in this study. Others report that NR supplementation increases the abundance of NAD^+^ in various model systems including cells (Schondorf et al., 2018) and mice (Cantó et al., 2012; Trammell et al., 2016; Schondorf et al., 2018; Yang et al., 2020). The tissue type the NAD^+^ measurements originated from is an important consideration when examining those studies. In mice, NAD^+^ flux experiments demonstrate that tissues metabolize NAD^+^ at different rates (Liu et al., 2018) and the natural abundance of NAD^+^ is tissue specific (Cantó et al., 2012; Liu et al., 2018). Furthermore, the ability of NR to increase NAD^+^ concentration appears to be tissue specific, with an increase in NAD^+^ after NR supplementation in mice identified in liver, skeletal muscle or brown adipose tissue, but not in white adipose tissue or brain (Cantó et al., 2012). In humans, NR supplementation is reported to increase NAD^+^ concentrations in the peripheral blood mononuclear cells of healthy older participants (Trammell et al., 2016; Martens et al., 2018), but not in the skeletal muscle of young (Stocks et al., 2021) or obese (Remie et al., 2020) subjects. Together, these studies indicate that tissue type influences the baseline NAD^+^ abundance and possibly the ability for NR to increase NAD^+^ concentrations.

The metabolomics analysis performed in this study was on whole flies, so it is not possible to determine the tissue or cellular origins of metabolites. By pooling all the tissues together, it is possible that tissue-specific changes in the abundance of NAD^+^ and its precursors due to NR supplementation were not detected.

NAD^+^ exists in three intracellular compartments: cytosolic, nuclear and mitochondrial (Cambronne and Kraus, 2020; Amjad et al., 2021). Elevated NAD^+^ abundance within these pools can stimulate key NAD^+^-consuming enzymes located within that compartment (Cambronne and Kraus, 2020; Amjad et al., 2021). NR supplementation stimulates SIRT1 and SIRT3 in mice (Cantó et al., 2012; Brown et al., 2014), which are located in the nucleus and mitochondria, respectively (Onyango et al., 2002; Michishita et al., 2005; Zakhary et al., 2010). Therefore, NR supplementation likely increases the abundance of NAD^+^ in the nuclear and mitochondrial compartment (Cantó et al., 2012; Cambronne and Kraus, 2020; Mehmel et al., 2020).

We demonstrate that Sir2 (homologous to mammalian SIR2/SIRT1) is required for the beneficial effects of NR in *Tafazzin* mutants. NR supplementation is likely increasing the abundance of NAD^+^ in the nuclear compartment. Measuring NAD^+^ abundance *in vivo* and without disturbing the physiological environment within the compartments is complicated, and current techniques are unable to distinguish between bound and free NAD^+^ (Cambronne and Kraus, 2020). Therefore, in this study, we did not measure the abundance of NAD^+^ within these intracellular compartments. Future work may help illuminate the dynamic interactions between the different NAD^+^ pools and SIRT1 activity in the context of Barth syndrome.

Metabolomics data show that overexpressing *spargel* in *Taz^889^* flies affects the NAD^+^:NADH ratio in whole flies more than NR supplementation does. The improved NAD^+^:NADH ratio is likely to be due to an increase in NADH oxidation because the abundance of NADH returns to wild-type levels, although there was no difference in the abundance of NAD^+^. This suggests that NADH is being more efficiently utilized in *Taz^889^* flies overexpressing *spargel* relative to *Taz^889^* flies fed NR.

NADH is oxidized by several enzymes, including lactate dehydrogenase, to replenish NAD^+^ during glycolysis and by complex I of the mitochondrial respiratory chain (Berrisford and Sazanov, 2009; Sazanov, 2015). There was no increase in NADH oxidation by lactate dehydrogenase, because there was no increase in lactate production between the experimental groups. Overexpression of *spargel* increases the number of mitochondria (Lehman et al., 2000; Tiefenböck et al., 2010) and the proteins of the electron transport chain (Wu et al., 1999). Therefore, owing to the increase in mitochondrial number, and probable increase in complex I proteins, it is likely that more NADH is being oxidized by mitochondria in *Taz^889^* flies overexpressing *spargel*, resulting in lower NADH content and improved mitochondrial coupling.

Disrupted CL remodeling is a key phenotype of Barth syndrome (Vreken et al., 2000). The most abundant CL species in control flies (64:4 and 66:5) were reduced in *Taz^889^* flies. The CL species 64:4 and 66:5 most likely contain the smaller fatty acid C16:1, which was also found to be the most abundant CL in wild-type flies (Xu et al., 2006). The MLCL:CL ratio is also important in the context of Barth syndrome because patients with a lower MLCL:CL ratio tend to have less-severe phenotypes (Bowron et al., 2015). Coinciding with human data (Bowron et al., 2015), *Taz^889^* flies overexpressing *spargel* have a reduced MLCL:CL ratio, and their exercise capacity is better than that of *Taz^889^* flies. MLCL can accumulate due to deacylation of CL during the remodeling processes or due to degradation of CL. The improved MLCL:CL ratio in *Taz^889^* flies overexpressing *spargel* is likely to be due to decreased CL degradation, because total MLCL did not change but the total CL did increase. This hypothesis is supported by previous results that also found that the MLCL:CL ratio improved in the flight muscles of flies overexpressing *spargel* (Xu et al., 2019). CL degradation is slowed when CL is associated with other membrane proteins (Xu et al., 2016, 2019). The degradation of CL is possibly delayed in *Taz^889^* flies overexpressing *spargel* due to an increase in mitochondrial membrane proteins (Kaufman et al., 2007; Ventura-Clapier et al., 2008).

In the context of a mitochondrial disease like Barth syndrome, it is reasonable to be cautious about increasing the number of poorly functioning mitochondria, with the possibility of increasing oxidative damage. However, upregulation of *spargel* also increases various antioxidant defense systems to neutralize oxidative species produced by increased oxidative phosphorylation (Austin and St-Pierre, 2012). The ratio between GSH and GSSG, which is a marker of oxidative stress (Kadiiska et al., 2000; Singh et al., 2012), was not significantly higher in *Taz^889^* flies when they overexpressed *spargel*, suggesting that if there is an elevated amount of oxidative stress, the amount is not enough to overwhelm the antioxidant mechanisms and cause substantial damage.

Muscle weakness is a diagnostic characteristic of Barth syndrome (Bittel et al., 2018), so reduced exercise capacity in a muscle-specific *Tafazzin* knockdown fly was expected. The improvement to endurance observed when *Tafazzin* was restored specifically in neurons is intriguing. Little is known about the role neurons play in the pathogenesis of Barth syndrome, although cognitive dysfunction has been reported in Barth patients (Mazzocco and Kelley, 2001; Mazzocco et al., 2007). Aging studies demonstrate a correlation between an altered CL profile due to oxidative damage and poor neuronal function (Sen et al., 2007; Lesnefsky and Hoppel, 2008; Petrosillo et al., 2008; Falabella et al., 2021). Even though mitochondria in the muscle are still dysfunctional in neuronal-specific *Tafazzin* rescue flies, the endurance and climbing speed are improved by day 12. Thus, restoration of *Tafazzin* in neurons restores baseline exercise capacity without modifying mitochondrial function within muscle tissue. Follow-up studies will be needed to determine what subset of neurons is responsible for this rescue phenotype and how this observation could be leveraged for future therapies.

To our surprise, knocking down *sir2* in *Taz^889^* flies increased endurance similarly to when *sir2* was overexpressed. However, the mitochondrial function was not restored in *Taz^889^* flies with *sir2* knocked down, whereas the RCR in *sir2* rescue flies was improved.

Knockdown of *sir2* in wild-type flies does not increase endurance. Considering our results together, we believe that knockdown of *sir2* in *Tafazzin* mutants likely improves endurance through a different mechanism than overexpressing *sir2.* The increase in the endurance of *Taz^889^* flies with *sir2* knocked down may occur because there is more available NAD^+^ to be utilized by other NAD-dependent enzymes. Although out of the scope of this study, investigation into these enzymes would be valuable as they could be another therapeutic target.

The therapeutic potential of NR, *SIR2* (*SIRT1*) and *PGC-1α* (*PPARGC1A*) for Barth patients is exciting. Other pharmacological stimulators of the NR–SIR2–PGC-1α axis could also have the potential to alleviate symptoms of Barth syndrome. This study demonstrates that NR supplementation provides enormous benefits to the exercise capacity of *Tafazzin* mutants, and that it works though *sir2* and *spargel.* We demonstrated that *sir2* and *spargel* do not need to be overexpressed throughout the whole body for benefits to occur, just in either muscle or neural tissue. Furthermore, overexpression of *sir2* or *spargel* in muscle or neurons can fully mimic restoration of *Tafazzin*. Although gene therapies are still being developed, supplementation with NR is a promising alternative therapy worthy of further investigation.

## MATERIALS AND METHODS

### *Drosophila* lines, maintenance and genetics

Flies were raised at 25°C with 50% humidity. Flies were fed a 10% yeast/sugar diet and kept on a 12-h light/dark cycle. Fly lines were obtained from the Bloomington *Drosophila* Stock Center (Bloomington, IN, USA), except the following: *Taz^889^* (WellGenetics, Taipei City, Taiwan); Δ*Taz*/Cyo (Mindong Ren, NYU Langone Health, New York, NY, USA); *uas-Tafazzin/Sb*, *ELAVGS-GAL4* (Mindong Ren, NYU Langone Health); *uas-srl* (David Walker, University of California, Los Angeles, Los Angeles, CA, USA).

Standard cross schemes were used to create the following lines: *Taz^889^; MHC-GS-Gal4*; *Taz^889^; ELAV-GS-Gal4*; *Taz^889^; TUB5-GS-Gal4*; *Taz^889^; uas-srl*; *Taz^889^; srl^1^*; *Taz^889^; uas-sir2* RNAi; *Taz^889^; uas-sir2*. *Taz^2^*, which is shown in Fig. 7 and Fig. S3, is a trans-heterozygous *Tafazzin* mutant with one *Taz^889^* allele and one Δ*Taz* allele (Xu et al., 2006).

‘GS’ denotes a gene-switch line that expresses the Gal4 protein when fed mifepristone (100 μM dissolved in 70% ethanol). Flies fed mifepristone are designated as ‘On’; flies fed the vehicle are designated as ‘Off’. Using this method controls for genetic background effects because the flies are isogenic, differing only in whether they received the inducing drug. Additionally, this method allows for all gene expression experiments to occur after adult eclosion to avoid any developmental phenotypes. Experimental flies collected within a 48-h period after eclosing were considered age matched. Flies were fed mifepristone at day 2 and were assessed at the earliest on day 5, allowing for at least 2 full days for induced gene expression changes to accumulate.

### Creation of the *Taz^889^* mutant using CRISPR/Cas9 genome editing

*Taz^889^* flies were created (WellGenetics) using CRISPR/Cas9-mediated genomic editing by homology-dependent repair. The 8th to 896th nucleotides of the *Tafazzin* gene were deleted because this size deletion produced a prominent phenotype in a previously published *Tafazzin* mutant (Xu et al., 2006). In place of the deletion, an RFP marker was knocked in to track the presence of the mutation through various cross schemes. All new transgenic experimental flies were ‘gene-switch’ lines and were compared to the identical background without the inducing drug. The CRISPR plasmid containing the deletion was injected into a *w^1118^* line. That progenitor line was assessed next to *Taz^889^* flies and served as a genetic background control. The line was validated by PCR and genomic sequencing. Sequencing and Blast results confirmed the deletion and RFP insertion.

#### Drug treatment

NR (TRU NIAGEN, ChromaDex, Los Angeles, CA, USA) was dissolved in water to create 1 mM concentration, unless stated otherwise. Then, 50 μl of the solution was applied to the surface of the food and allowed to dry. Flies were placed onto the food at day 4 and allowed 5 full days on the drug food before assessments.

#### Two-phase extraction metabolomics and lipidomics analyses

Metabolomics and lipidomics analyses were performed in 2-5 mg of freeze-dried flies following a two-phase extraction method (Molenaars et al., 2021). Samples were homogenized using a TissueLyser II device (Qiagen; 5 min at 30 pulses/s) in 425 µl water, 500 µl methanol and 175 µl internal standards mixture. After homogenization, 1000 µl chloroform was added, and samples were thoroughly mixed and centrifuged (5 min, 16,000 ***g***, 4°C), creating a two-phase system. The top polar phase was transferred to clean tubes and dried in a vacuum evaporator at 60°C. The bottom apolar fraction was transferred to glass vials and evaporated under a stream of nitrogen at 45°C.

For metabolomics analysis, pellets obtained after evaporation of the polar phase were dissolved in 100 µl methanol/water (6/4; v/v) and analyzed in an Aquity UPLC system (Waters, Milford, MA, USA), coupled to an Impact II Ultra-High Resolution Qq-Time-Of-Flight MS (Bruker, Billerica, MA, USA). Metabolites were chromatographically separated in a SeQuant ZIC-cHILIC column (PEEK 100×2.1 mm, 3 µm particle size; Merck, Darmstadt, Germany) at 30°C using a method consisting of a gradient running at 0.25 ml/min from 100% mobile phase B (9:1 acetonitrile:water containing 5 mM ammonium acetate pH 6.8) to 100% mobile phase A (1:9 acetonitrile:water containing 5 mM ammonium acetate pH 6.8) for 28 min, followed by a re-equilibration step at 100% B for 5 min. Mass spectrometry (MS) data were acquired in negative and positive ionization modes over the range of 50-1200 m/z and analyzed using Bruker TASQ software (version 2.1.22.1 1065).

For lipidomics analysis, after evaporation of the apolar fraction, pellets were dissolved in 150 µl chloroform/methanol (1/1; v/v) and analyzed in a Thermo Fisher Scientific Ultimate 3000 binary UPLC coupled to a Q Exactive Plus Orbitrap mass spectrometer using nitrogen as a nebulizing gas. The spray voltage was set at 2500 V, and the capillary temperature was set at 256°C. For normal-phase separation, a Phenomenex^®^ LUNA silica, 250×2 mm, 5 µm 100 Å, was used. Mobile phase consisted of (A) 85:15 (v/v) methanol:water containing 0.0125% formic acid and 3.35 mM ammonia and (B) 97:3 (v/v) chloroform:methanol containing 0.0125% formic acid. The LC gradient consisted of 10% A for 0-1 min, reach 20% A at 4 min, reach 85% A at 12 min, reach 100% A at 12.1 min, 100% A for 12.1-14 min, reach 10% A at 14.1 min, 10% A for 14.1-15 min at a flow rate of 0.3 ml/min and a column temperature of 25°C. For reverse-phase separation, a Waters HSS T3 column, 150×2.1 mm, 1.8 µm, was used. Mobile phase consisted of (A) 4:6 (v/v) methanol:water and (B) 1:9 (v/v) methanol:isopropanol, containing 0.1% formic acid and 10 mM ammonia in both cases. The LC gradient consisted of 100% A at 0 min, reach 80% A at 1 min, reach 0% A at 16 min, 0% A for 16-20 min, reach 100% A at 20.1 min, 100% A for 20.1-21 min at a flow rate of 0.4 ml/min and a column temperature of 60°C. MS data were acquired using negative and positive ionization using continuous scanning over the range of 150-2000 m/z. Data were analyzed using an in-house-developed metabolomics pipeline written in the R programming language (Molenaars et al., 2021).

All reported metabolite and lipid abundances were normalized to total dry weight as well as to internal standards with comparable retention times and response in the MS.

#### Endurance

The endurance of flies was measured as previously described (Tinkerhess et al., 2012a; Damschroder et al., 2018a). Eight vials (*n*=20 flies) from each cohort were placed onto the Power Tower machine, which stimulates flies to run upwards within their vial until they are physically fatigued. When 80% of the flies within a vial had stopped climbing a minimum of one body length upwards, the vial was removed, and the time was recorded. Survival curves were created in GraphPad Prism (San Diego, CA, USA), and significance was determined using log-rank tests. All assessments were repeated in either duplicate or triplicate. All endurance graphs in the figures show complete data from one repetition and show the same statistical differences as the other replicates unless otherwise stated.

#### Climbing speed

Climbing speed was measured by performing the rapid iterative negative geotaxis (RING) assessment (Gargano et al., 2005). Flies were moved to a clear polypropylene vial (*n*=100 flies total, 20 flies per vial, five vials), and allowed to acclimate for 1 min. Vials were moved to a RING apparatus, which allows for five vials to be moved at once. Flies were tapped down to the bottom of their vials and allowed 2 s to climb upwards. After 2 s, a picture was taken to capture their climbing height. Four pictures were taken per group.

ImageJ (National Institutes of Health, Bethesda, MD, USA) was used to quantify the height climbed in 2 s, expressed in quadrants. The average of the four pictures was calculated and plotted. Significance was determined using an unpaired two-tailed Student’s *t*-test. The climbing speed of at least two separate cohorts was assessed, with 100 flies per cohort. One representative repetition is displayed.

#### Mitochondrial isolation

Isolated mitochondria from 60 flies were used for each biological replicate. Flies were anesthetized on ice for 1 min. When heads and thoraces were analyzed, 70-80 fly heads and thoraces were collected. Carcasses were placed in 500 μl isolation buffer [0.32 M sucrose, 10 mM EDTA, 10 mM Tris-HCl, 2% fatty acid-free bovine serum albumin (BSA), pH 7.3] (Ferguson et al., 2005). Flies were homogenized using a glass-Teflon Dounce homogenizer (Ferguson et al., 2005; Holmbeck et al., 2015). The homogenate was then filtered through a nylon filter (sigma; pore size, 10 μm) (Ferguson et al., 2005), and the filter was washed with 1 ml isolation buffer. The homogenate was centrifuged at 300 ***g*** for 5 min at 4°C (Holmbeck et al., 2015). Then, the supernatant was moved to a new tube and centrifuged at 6000 ***g*** for 10 min at 4°C. The mitochondrial-enriched pellet was resuspended in 30-50 μl respiration buffer (120 mM KCL, 5 mM KH_2_PO_4_, 3 mM HEPES, 1 mM EGTA, pH 7.2, BSA free) (Ferguson et al., 2005), and protein concentration was determined using the BCA assay (Thermo Fisher Scientific, Rockford, IL, USA). Mitochondrial respiration of isolated mitochondria was measured within 2 h of isolation.

#### Mitochondrial respiration

Respiration rates were obtained using a Clark-type electrode (Hansatech Instruments, Norfolk, UK). For all experiments, the chamber temperature was 25°C, and 10 μl of isolated mitochondria was added to 990 ml of respiration buffer supplemented with 0.3% BSA. The substrates pyruvate and malate were added to the chamber for a final concentration for 10 μM. State III respiration was induced by addition of ADP (125 nmol) and state IV was induced by adding oligomycin (2.5 μM). The respiratory control ratio was calculated from the respiration rates of state III to state IV. Two technical repetitions were performed for each biological replicate. In total, six biological replicates were analyzed and graphed. Statistical significance was determined using either an unpaired two-tailed Student’s *t*-test or a two-way ANOVA with post-hoc Tukey multiple comparison when appropriate. A *P*-value less than 0.05 was considered significant.

### qRT-PCR

Gene expression was confirmed by qRT-PCR. Three independent biological replicates were examined with three technical repetitions performed for each. To confirm the RNAi efficacy in the heart-specific and fat body-specific *Tafazzin* knockdown, cDNA was isolated from 20 adult fly hearts and ten adult fly fat bodies (Sujkowski et al., 2020), using a Cells-to-CT Kit (Invitrogen, Waltham, MA, USA). Relative abundance of *Tafazzin* was determined by amplification and staining with SYBR GREEN and measuring florescence using a StudioQuant 3 Real-time PCR System (Thermo Fisher Scientific). For all other genotypes, total RNA or RNA from either the heads or thoraces of flies was isolated using TRIZOL (Invitrogen). One-step qRT-PCR was performed using Power SYBR Green Master Mix (Applied Biosystems, Waltham, MA, USA) and a StudioQuant 3 Real-time PCR System (Thermo Fisher Scientific). Each 20 μl reaction contained RNA (4 μl, 25 ng/μl), forward and reverse primers (2 μl, 1 μM), Power SYBR Green PCR Master Mix (10 μl), reverse transcriptase (0.1 μl), inhibitor (0.025 μl) and dH_2_O (3.875 μl). The quantitative PCR (qPCR) program ran for 40 cycles of 95°C for 15 s followed by 60°C for 1 min. mRNA data were normalized to *Act5C.*

Primers used were as follows: *Act5C* F, 5′-CGCAGAGCAAGCGTGGTA-3′; *Act5C* R, 5′-GTGCCACACGCAGCTCAT-3′; *Tafazzin* F, 5′-CGTGTGTTCCAATTTAAGGCGT-3′; *Tafazzin* R, 5′-AGTCTGGGTGGCGATATCCT-3′; *srl* F, 5′-GGATTCACGAATGCTAAATGTGTTCC-3′; *srl* R, 5′-GATGGGTAGGATGCCGCTCAG-3′; *sir2* F, 5′-GCCTCCAGGACAGTTAGCAG-3′; *sir2* R, 5′-GCCAATCTCTTGTTCTTCCC-3′.

### mtDNA copy number

DNA was isolated from 20 flies using phenol-chloroform (Sigma Aldrich, St Louis, MO, USA) (*n*=20 flies per replicate group). mtDNA was quantified by amplifying *lrRNA*, which does not have a nuclear copy in *Drosophila melanogaster* (Correa et al., 2012), and nuclear genomic DNA was quantified by amplifying *rp2* (Correa et al., 2012).

qPCR (Aw et al., 2018) was performed using an ABI 7300 Real Time PCR System (Applied Biosystems) and Power SYBR Green PCR Master Mix (Applied Biosystems). The qPCR program started with a denaturing step at 95°C for 5 min followed by amplification for 40 cycles that consisted of 95°C for 5 min followed by 60°C for 30 s (Aw et al., 2018). The mtDNA copy number was expressed as the average fold change of mtDNA to nuclear DNA (Aw et al., 2018). Primers were previously published (Correa et al., 2012). The data were analyzed using either an unpaired two-tailed Student's *t*-test or a two-way ANOVA with post-hoc Tukey multiple comparison.

Primer sequences were as follows: *lrRNA* F, 5′-TCGTCCAACCATTCATTCC-3′; *lrRNA* R, 5′-ATAAAGTCTAACCTGCCCACTGA-3′; *rp2* F, 5′-AGGCGTTTGAGTGGTTGG-3′; *rp2* R, 3′-TGGAAGGTGTTCAGTGTCAT-3′.

## Supplementary Material

10.1242/dmm.049279_sup1 Supplementary information
