# Correction: Stimulating the *sir2–spargel* axis rescues exercise capacity and mitochondrial respiration in a *Drosophila* model of Barth syndrome

**DOI:** 10.1242/dmm.052040

**Published:** 2024-09-26

**Authors:** Deena Damschroder, Rubén Zapata-Pérez, Kristin Richardson, Frédéric M. Vaz, Riekelt H. Houtkooper, Robert Wessells

There was an error published in *Dis. Model. Mech.* **15**, dmm049279 (doi:10.1242/dmm.049279).

An incorrect version of Fig. 2 was published in which panels G-I had accidentally been deleted. The corrected and original Fig. 2 are shown below. Both the online full-text and PDF versions of the article have been updated with the correct figure.

DMM and the authors apologise for this error and any inconvenience it may have caused.

**Fig. 2 (corrected).**
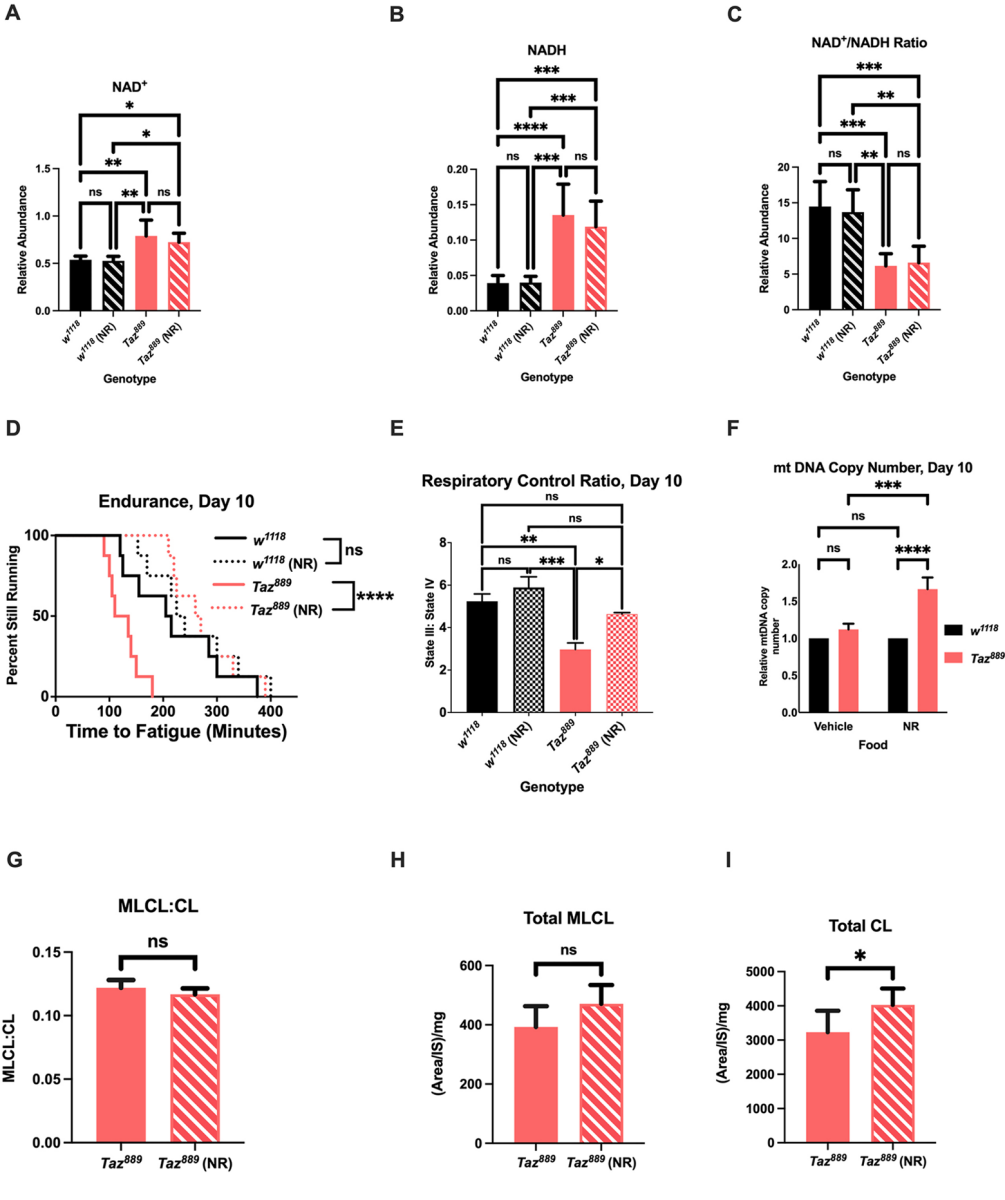
**Nicotinamide riboside (NR) supplementation provides benefits to *Tafazzin* mutants.** (A,B) The abundance of NAD^+^ (B) and NADH (A) was measured by mass spectrometry and normalized to total protein levels (six biological repetitions with six flies per repetition, data are mean±s.d., two-way ANOVA, genotype effect, *P*<0.0001, Tukey post-hoc test). (C) The NAD^+^:NADH ratio was calculated from the abundances for those molecules (data are mean±s.d., two-way ANOVA, genotype effect, *P*<0.0001, Tukey post-hoc test). (D,E) NR supplementation restores the endurance of *Taz^889^* flies (log-rank analysis, *n*=8 vials, 20 flies per vial; D) and the respiratory control ratio (RCR) (six biological replicates, data are mean±s.e.m., *n*=60 per replicate, genotype effect, *P*<0.0001, NR effect, *P*=0.0029, Tukey post-hoc test; E). (F) At day 10, the relative mitochondrial DNA (mtDNA) copy number is not different between *Taz^889^* and control flies, but NR supplementation increases mtDNA in *Taz^889^* flies (*n*=3 biological replicates, data are mean±s.d., two-way ANOVA, genotype effect, *P*<0.0001, NR effect, *P*=0.0006, Tukey post-hoc test). (G) Feeding NR to *Taz^889^* flies does not alter the MLCL:CL ratio (unpaired two-tailed Student's *t-*test, six biological repetitions with six flies per repetition). (H,I) There is no difference in total MLCL (H) between *Taz^889^* flies with and without NR feeding, but NR-fed *Taz^889^* flies have an increased abundance of total CL (I) (unpaired two-tailed Student's *t-*test, six biological repetitions with six flies per repetition). *P<0.05, **P<0.01, ***P<0.001, ****P<0.0001; ns, not significant.

**Fig. 2 (original).**
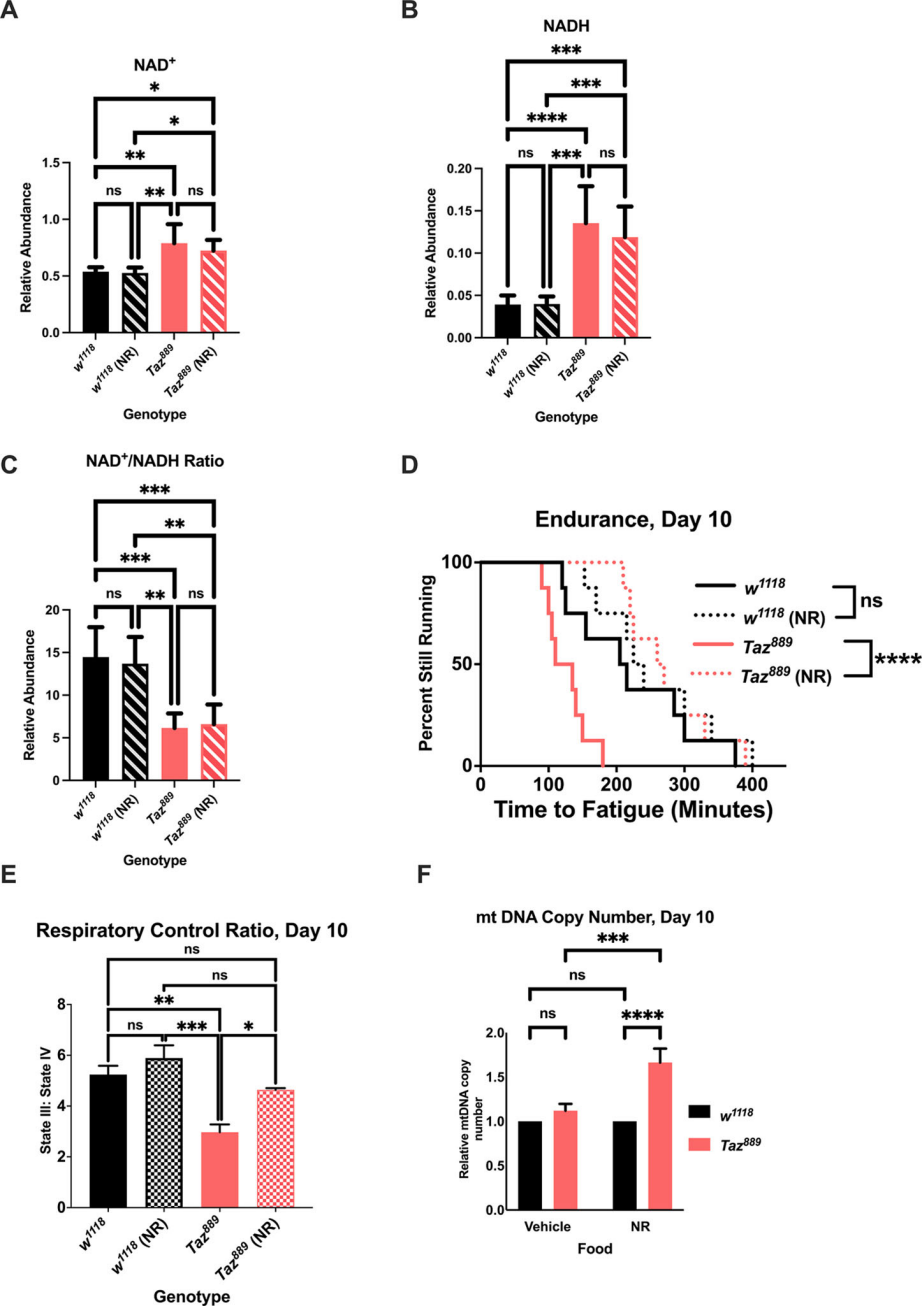
**Nicotinamide riboside (NR) supplementation provides benefits to *Tafazzin* mutants.** (A,B) The abundance of NAD^+^ (B) and NADH (A) was measured by mass spectrometry and normalized to total protein levels (six biological repetitions with six flies per repetition, data are mean±s.d., two-way ANOVA, genotype effect, *P*<0.0001, Tukey post-hoc test). (C) The NAD^+^:NADH ratio was calculated from the abundances for those molecules (data are mean±s.d., two-way ANOVA, genotype effect, *P*<0.0001, Tukey post-hoc test). (D,E) NR supplementation restores the endurance of *Taz^889^* flies (log-rank analysis, *n*=8 vials, 20 flies per vial; D) and the respiratory control ratio (RCR) (six biological replicates, data are mean±s.e.m., *n*=60 per replicate, genotype effect, *P*<0.0001, NR effect, *P*=0.0029, Tukey post-hoc test; E). (F) At day 10, the relative mitochondrial DNA (mtDNA) copy number is not different between *Taz^889^* and control flies, but NR supplementation increases mtDNA in *Taz^889^* flies (*n*=3 biological replicates, data are mean±s.d., two-way ANOVA, genotype effect, *P*<0.0001, NR effect, *P*=0.0006, Tukey post-hoc test). (G) Feeding NR to *Taz^889^* flies does not alter the MLCL:CL ratio (unpaired two-tailed Student's *t-*test, six biological repetitions with six flies per repetition). (H,I) There is no difference in total MLCL (H) between *Taz^889^* flies with and without NR feeding, but NR-fed *Taz^889^* flies have an increased abundance of total CL (I) (unpaired two-tailed Student's *t-*test, six biological repetitions with six flies per repetition). *P<0.05, **P<0.01, ***P<0.001, ****P<0.0001; ns, not significant.

